# Local adaption of intrapartum clinical guidelines, United Republic of Tanzania

**DOI:** 10.2471/BLT.18.220830

**Published:** 2019-03-26

**Authors:** Nanna Maaløe, Tarek Meguid, Natasha Housseine, Britt Pinkowski Tersbøl, Karoline Kragelund Nielsen, Ib Christian Bygbjerg, Jos van Roosmalen

**Affiliations:** a Global Health Section, Department of Public Health, University of Copenhagen, Øster Farimagsgade 5, Building 9, 1353 Copenhagen, Denmark.; b Mnazi Mmoja Hospital, Zanzibar, United Republic of Tanzania.; c Health Promotion Research, Steno Diabetes Center Copenhagen, Gentofte, Denmark.; d Athena Institute, Vrije Universiteit Amsterdam, Amsterdam, Netherlands.

## Abstract

**Problem:**

Gaps exist between internationally derived clinical guidelines on care at the time of birth and realistic best practices in busy, low-resourced maternity units.

**Approach:**

In 2014–2018, we carried out the PartoMa study at Zanzibar’s tertiary hospital, United Republic of Tanzania. Working with local birth attendants and external experts, we created easy-to-use and locally achievable clinical guidelines and associated in-house training to assist birth attendants in intrapartum care.

**Local setting:**

Around 11 500 women gave birth annually in the hospital. Of the 35–40 birth attendants employed, each cared simultaneously for 3–6 women in labour. At baseline (1 October 2014 to 31 January 2015), there were 59 stillbirths per 1000 total births and 52 newborns with an Apgar score of 1–5 per 1000 live births. Externally derived clinical guidelines were available, but rarely used.

**Relevant changes:**

Staff attendance at the repeated trainings was good, despite seminars being outside working hours and without additional remuneration. Many birth attendants appreciated the intervention and were motivated to improve care. Improvements were found in knowledge, partograph skills and quality of care. After 12 intervention months, stillbirths had decreased 34% to 39 per 1000 total births, while newborns with an Apgar score of 1–5 halved to 28 per 1000 live births.

**Lessons learnt:**

After 4 years, birth attendants still express high demand for the intervention. The development of international, regional and national clinical guidelines targeted at low-resource maternity units needs to be better attuned to input from end-users and the local conditions, and thereby easier to use effectively.

## Résumé

**Problème:**

Des écarts existent entre les directives cliniques internationales relatives aux soins au moment de la naissance et les meilleures pratiques réalistes dans les services de maternité très sollicités et à faibles ressources.

**Approche:**

En 2014-2018, nous avons réalisé l'étude PartoMa au sein de l'hôpital tertiaire de Zanzibar, en République-Unie de Tanzanie. Dans le cadre d'une collaboration avec des professionnels locaux de l'accouchement et des experts externes, nous avons créé des directives cliniques faciles à utiliser et applicables localement, et nous avons associé à ces directives une formation interne destinée à aider les professionnels de l'accouchement lors des soins pendant l'accouchement.

**Environnement local:**

Environ 11 500 femmes accouchent chaque année dans cet hôpital. Chacun des 35-40 professionnels de l'accouchement employés s'occupait simultanément de 3 à 6 femmes en travail. Au début de l'étude (du 1er octobre 2014 au 31 janvier 2015), on dénombrait 59 mortinaissances pour 1 000 naissances totales et 52 nouveaux nés avec un score d'Apgar de 1-5 pour 1 000 naissances vivantes. Des directives cliniques provenant de l'extérieur étaient disponibles, mais rarement utilisées.

**Changements significatifs:**

La participation du personnel aux différentes formations était bonne, alors que les séminaires étaient organisés en dehors des heures de travail et ne donnaient pas lieu à une rémunération supplémentaire. De nombreux professionnels de l'accouchement ont apprécié l'intervention et étaient désireux d'améliorer les soins apportés. Des améliorations ont été constatées concernant les connaissances, l'utilisation du partogramme et la qualité des soins. Après 12 mois d'intervention, le taux de mortinatalité avait diminué de 34%, passant à 39 mortinaissances pour 1 000 naissances totales, tandis que le nombre de nouveaux nés avec un score Apgar de 1-5 avait diminué de moitié, passant à 28 pour 1 000 naissances vivantes.

**Leçons tirées:**

Après 4 ans, les professionnels de l'accouchement étaient toujours nombreux à vouloir participer à l'intervention. Les avis des utilisateurs finaux et les conditions locales doivent être mieux pris en compte lors de l'élaboration de directives cliniques internationales, régionales et nationales destinées aux services de maternité à faibles ressources pour être plus faciles à utiliser de façon efficace.

## Resumen

**Situación:**

Existen lagunas entre las directrices clínicas internacionales sobre la atención durante el parto y las mejores prácticas realistas en las unidades de maternidad concurridas y con escasos recursos.

**Enfoque:**

Entre 2014 y 2018, se llevó a cabo el estudio PartoMa en el hospital de nivel terciario de Zanzíbar, República Unida de Tanzania. Con la ayuda de asistentes de partos locales y expertos externos, se elaboraron directrices clínicas fáciles de usar y factibles localmente y se impartió formación interna asociada para ayudar a los asistentes de partos en la atención durante el parto.

**Marco regional:**

Alrededor de 11 500 mujeres dan a luz anualmente en el hospital. De los 35 a 40 asistentes de partos empleados, cada uno se ocupó simultáneamente de 3 a 6 mujeres de parto. Al inicio (del 1 de octubre de 2014 al 31 de enero de 2015), había 59 mortinatos por cada 1000 nacimientos totales y 52 recién nacidos con una puntuación de Apgar de entre 1 y 5 por cada 1000 nacidos vivos. Se disponía de directrices clínicas derivadas de fuentes externas, pero rara vez se utilizaban.

**Cambios importantes:**

La asistencia del personal a los repetidos cursos de formación fue buena, a pesar de que los seminarios se celebraron fuera del horario laboral y sin remuneración adicional. Muchos asistentes de partos apreciaron la intervención y se sintieron motivados para mejorar la atención. Se observaron mejoras en los conocimientos, las habilidades de partografía y la calidad de la atención. Después de 12 meses de intervención, los mortinatos disminuyeron un 34 % a 39 por cada 1000 nacimientos totales, mientras que los recién nacidos con una puntuación de Apgar de entre 1 y 5 se redujeron a la mitad a 28 por cada 1000 nacidos vivos.

**Lecciones aprendidas:**

Después de 4 años, los asistentes de partos todavía expresan una alta demanda de la intervención. La elaboración de directrices clínicas internacionales, regionales y nacionales dirigidas a las unidades de maternidad con escasos recursos debe estar mejor adaptada a las aportaciones de los usuarios finales y a las condiciones locales y, por tanto, ser más fácil de utilizar de manera eficaz.

## ملخص

**المشكلة:**

توجد فجوات بين المبادئ التوجيهية السريرية الدولية الخاصة الرعاية في وقت الولادة، وأفضل الممارسات الواقعية في وحدات الأمومة المزدحمة منخفضة الموارد.

**الأسلوب:**

قمنا في الفترة من 2014 إلى 2018، بإجراء دراسة PartoMa في مستشفى زنزبار فوق الثانوي، بجمهورية تنزانيا المتحدة. بناء على التعاون مع قابلات الولادة المحليات والخبراء الخارجيين، قمنا بوضع مبادئ توجيهية تتميز بسهولة الاستخدام وقابلة التحقيق المحلي، كما أضفنا التدريب الداخلي لمساعدة القابلات في الرعاية أثناء الولادة.

**المواقع:**

**المحلية ** تلد قرابة 11500 امرأة سنويًا في المستشفى. تم تعيين 35 إلى 40 قابلة للولادة، وكل منهن كانت تعتني بـ 3 إلى 6 سيدات أثناء وقت الولادة. في الأساس (من 1 تشرين أول/أكتوبر 2014 إلى 31 كانون ثاني/يناير 2015)، كانت هناك 59 حالة وفاة رُضّع لكل 1000 حالة ولادة و52 مولوداً بدرجة 1 إلى 5 على مقياس Apgar لكل 1000 حالة ولادة على قيد الحياة. كانت المبادئ التوجيهية السريرية الدولية متوفرة، ولكنها نادرة الاستخدام.

**التغييرات:**

**ذات** **الصلة ** كان معدل حضور فرق العمل في الدورات التدريبية المتكررة جيداً، بالرغم من أن الندوات كانت تُقام بعد ساعات العمل ودون أجر إضافي. أثنت العديدات من قابلات الولادة على هذا التدخل وكان لديهن الدافع لتحسين الرعاية. كانت هناك تحسينات واضحة في كل من المعرفة، ومهارات مخطط المخاض، وجودة الرعاية. بعد 12 شهراً من التدخل، انخفض معدل وفيات الرُضّع بمعدل 34% ليصبح 39 حالة لكل 1000 حالة ولادة، بينما وصل معدل المواليد الجدد بدرجة 1 إلى 5 على مقياس Apgar إلى النصف ليكون 28 حالة لكل 1000 حالة ولادة على قيد الحياة.

**الدروس:**

**المستفادة ** بعد مرور 4 أعوام، مازالت القابلات يعربن عن ارتفاع الطلب على التدخل. إن تطوير المبادئ التوجيهية الإكلينيكية الوطنية والإقليمية والدولية التي تستهدف وحدات الأمومة منخفضة الموارد، يحتاج إلى مواءمة أفضل من المستخدمين النهائيين والظروف المحلية، وبالتالي تكون أسهل في الاستخدام الفعال.

## 摘要

**问题:**

国际上制定的分娩护理临床指南与在繁忙、资源匮乏的妇产科医院实现最佳实践间存在差距。

**方法:**

2014–2018 年间，我们在坦桑尼亚联合共和国桑给巴尔三级医院进行了 PartoMa 研究。我们与当地接生人员和外部专家合作制定了易于使用、在当地可实行的临床指南以及相关的内部培训，以协助助产士进行分娩护理。

**当地状况:**

每年约有 11500 名妇女在这家医院分娩。在受雇的 35 至 40 名助产士中，每名助产士需同时照护 3 至 6 名产妇。在基准值上（2014 年 10 月 1 日至 2015 年 1 月 31 日），每 1000 名新生儿中有 59 名死胎，每 1000 名活产儿中有 52 名新生儿 Apgar 评分在 1–5 分。虽然外部制定的临床指南切实可行，但却很少使用。

**相关变化:**

尽管研讨会是在工作之余举办，并无额外报酬，但员工再培训的出勤率依然很高。许多助产士对此干预方式予以理解，并积极改善护理。知识水平、分娩技能和护理质量方面均有所改善。经过 12 个月的干预后，死胎率下降了 34%，每 1000 名新生儿中只有 39 名死胎，每 1000 名活产儿中仅有 28 名新生儿 Apgar 评分在 1–5 分，减少了近一半。

**经验教训:**

4 年过去了，助产士对干预的要求依然很高。制定针对资源匮乏的妇产科医院的国际、区域和国家临床指南需要更好地协调最终用户的投入和当地条件之间的关系，因而更容易有效使用。****

## Резюме

**Проблема:**

Существует разрыв между международными клиническими рекомендациями по уходу за матерью и ребенком в родах и практическим опытом, реализуемым в перегруженных и не располагающих достаточными ресурсами родильных отделениях.

**Подход:**

В 2014–2018 годах авторы провели исследование PartoMa в специализированной больнице Занзибара (Объединенная Республика Танзания). Работая с местными акушерами и сторонними специалистами, авторы создали простые в использовании и реально достижимые на местах клинические рекомендации и соответствующий учебный курс для акушеров по медицинскому обслуживанию в родах.

**Местные условия:**

Ежегодно в больнице рожают около 11 500 женщин. Из работающих 35–40 акушеров на каждого специалиста приходится одновременно от 3 до 6 рожениц. В качестве базового показателя (по состоянию с 1 октября 2014 года по 31 января 2015 года) было зафиксировано 59 случаев мертворождения на 1000 родов и 52 новорожденных с оценкой 1–5 по шкале Апгар на 1000 живорожденных младенцев. Клинические рекомендации, разработанные сторонними организациями, были доступны, но редко применялись.

**Осуществленные перемены:**

Несмотря на то что семинары проводились в нерабочее время и не оплачивались дополнительно, сотрудники исправно посещали многократные курсы обучения. Многие акушеры приветствовали принимаемые меры и были заинтересованы в совершенствовании качества медицинской помощи. Было отмечено улучшение знаний, навыков использования партографа и качества оказываемой помощи. Спустя 12 месяцев после начала вмешательства количество мертворождений снизилось на 34% (до 39 случаев на 1000 рождений), а количество новорожденных с оценкой 1–5 по шкале Апгар уменьшилось вдвое (до 28 случаев на 1000 живорожденных младенцев).

**Выводы:**

Спустя 4 года акушеры по-прежнему заинтересованы во вмешательстве в их сферу деятельности. При разработке международных, национальных и региональных клинических рекомендаций для родильных отделений, не располагающих достаточными ресурсами, следует более внимательно относиться к пожеланиям тех, кто будет этими рекомендациями пользоваться, и к местным условиям. В таком случае они будут использоваться более эффективно.

## Introduction

To end preventable deaths during birth, the production of clinical guidelines for low-resource settings is expanding.^1^ However, in many low- and middle-income countries, there are gaps between internationally derived clinical guidelines and realistic best practices. Therefore, guidelines tailored to local contexts are needed.^1^^–^^3^ We describe the PartoMa intervention study in Zanzibar, United Republic of Tanzania, for which a pocket guide on locally achievable maternity care was co-created with birth attendants. Examples of such adaptation and implementation of clinical guidelines in low-resource settings are scarce, both within and beyond maternal health.^1^^–^^3^

## Local setting

Mnazi Mmoja hospital is a low-resource tertiary hospital with a high burden of maternal and perinatal deaths. Around 11 500 women give birth annually in the hospital, with the great majority being self-referrals. At baseline (1 October 2014 to 31 January 2015), there were 59 stillbirths per 1000 total births and 52 newborns with an Apgar score of 1–5 per 1000 live births.^4^ The birth attendants are primarily young, non-specialized nurse-midwives and doctors, and staff turnover is high. Two years after the start of the intervention, 9 of 11 doctors (82%) and 15 of 24 nurse-midwives (63%) in permanent positions were no longer working in the maternity unit. Each birth attendant cares for between three to six women in labour at the same time, with, typically, two women sharing each bed.

When starting the study in October 2014, different clinical guidelines on intrapartum care, including the World Health Organization’s (WHO’s) *Guidelines for managing complications in pregnancy and childbirth*,^5^ were available in the hospital, but rarely used.^4^

## Approach

The aim was to create locally achievable and easy-to-use guidelines to assist birth attendants in providing best possible intrapartum care with the constrained resources available. We adapted existing international guidelines,^5^^–^^7^ and we conducted systematic literature searches when we had to deviate from the recommendations to reach reality. Our modifications included reducing the frequency of clinical assessments, reducing the information load and avoiding ambiguity within recommendations (Box 1). As described elsewhere,^2^ the development process included repeated reviews by local birth attendants and by a panel of external specialists in midwifery, obstetrics and neonatology, with experience from low-resource settings. We applied three key principles: (i) guidelines for individual women should consider that each birth attendant cared for several women in labour simultaneously; (ii) basic and emergency obstetric care should be integrated; and (iii) the WHO partograph should be emphasized as an early warning tool to assess maternal condition, fetal heart rate and progression throughout the latent and active phases of labour.

Box 1 **Examples of reflections in the development of intrapartum clinical guidelines at Zanzibar’s tertiary hospital, United Republic of Tanzania**

**1. Creating time for managing complications**

We considered how birth attendants could allocate more time to attend to women with labour complications, such as slow labour progress, while still providing essential monitoring and care for women with uncomplicated labour. Following the World Health Organization (WHO) 2017 recommendations,^5^ basic assessments of fetal heart rate, contractions, blood pressure, pulse and vaginal examination during four hours of uncomplicated first stage of active labour, would take 110 minutes per woman. Notably, this excludes additional time for providing caring support. As one birth attendant at Zanzibar’s tertiary hospital typically cared simultaneously for three or more women in labour, the birth attendant was inevitably forced to prioritize even these basic tasks. Adding to the challenge, levels of knowledge, skill and experience on basic obstetric care were limited among birth attendants, which could compromise attendants’ ability to prioritize care in complex and hectic care situations. To create time for managing complications and assist in prioritization, the PartoMa guidelines therefore recommended a lower frequency of basic assessments. This allowed a minimum of 33 minutes per woman for basic assessments over four hours.

**2. Safe management of slow labour progress**

Blind titration of intravenous oxytocin during labour may cause uterine hyperstimulation with risk of fetal death, uterine rupture and bleeding after birth. Cautious augmentation of labour with oxytocin is thus warranted, but is often hampered in busy, low-resource maternity units by lack of one-to-one care and electronic drip-count devices. WHO recommendations from 2017^5^ are that augmentation may be started when cervical dilatation is less than 1 cm per hour. This would cause overuse among many women with uncomplicated variations in the progress of labour. The guidance is, however, contradicted by other WHO recommendations in 2014 and 2018,^6^^,^^7^ both of which endorse an undefined interval of watchful waiting before starting oxytocin. In Zanzibar’s tertiary hospital, considering the high workload carried by too-few birth attendants, and attendants’ limited knowledge and skills, we decided it was dangerous and unfair to leave the birth attendants alone to judge how long an undefined interval may last. The PartoMa guidelines therefore recommended reserving intrapartum oxytocin augmentation for women crossing the partograph’s action line indicating severe prolonged labour.

Note: The PartoMa pocket guide is available online at https://publichealth.ku.dk/about-the-department/global/research/sexual-and-reproductive-health-and-rights/partoma/dokumenter/PartoMa_Pocket_Guide_2_22062018.pdf.

In addition to everyday use, training on the use of the pocket guide and partograph was provided during quarterly in-house seminars.^8^ We ran each 4-hour seminar twice to enable most staff to participate, and participants were invited to return for the following rounds of quarterly seminars. Participants included nurse midwives, doctors, assisting medical officers, clinical officers and students. Group sizes varied between five and 15, as no staff were refused access. No allowances were paid, but food, pocket guides and certificates of attendance were provided. The main focus was to strengthen critical clinical decision-making by use of the clinical guidelines, teamwork and triage, as well as strengthening respectful caring support of women in labour. At each seminar, the groups rotated between five stations, where real case stories from the hospital were discussed. The case stories differed between the quarterly seminar rounds to ensure relevance among returnees, but one station always focused on triage and the others on individual case stories. The facilitators were experienced birth attendants, predominantly from the hospital.

In response to birth attendants’ comments, we launched an expanded second version of the pocket guide in 2018. Though the official work language is English, we are currently translating the guide into Swahili, which might strengthen use further.^8^

## Relevant changes 

We evaluated the PartoMa intervention using Kirkpatrick’s four-level framework.^9^ Level 1 was staff perceptions, which we evaluated throughout the four years. Since January 2015, 16 seminar rounds have been conducted at the hospital. During the first 12 rounds, an average of 67 birth attendants (range: 45–120) participated in each round, with approximately 40% returnees. From the hospital’s maternity unit, an average of 7 doctors (64%) and 15 nurse midwives (62%) attended each time, as well as nearly all intern doctors (range: 4–14). Additional participants came from other maternity units in Zanzibar. Among 532 participants who responded to anonymous seminar evaluation questionnaires, 484 (91%) would recommend the seminars to fellow birth attendants, 458 (86%) agreed that the case stories represented familiar clinical situations and 484 (91%) believed that the seminars and pocket guides improved intrapartum care.

Level 2 was birth attendants’ knowledge and partograph skills, which we evaluated during December 2017 to June 2018. Paired comparisons before and after seminars showed significant improvements in knowledge and partograph skills among 143 seminar participants, of whom 24 were followed through an additional seminar round where further significant improvements were found (Nanna M. and Thomsen C., Mnazi Mmoja Hospital, unpublished data, October 2018).

Levels 3 and 4 were clinical practice and birth outcomes. We conducted a pre–post-intervention study, comparing case files at baseline (October 2014 to January 2015) and one year later (October 2015 to January 2016). As previously published,^10^^,^^11^ we found multiple significant improvements in quality of care, such as reduced median time from last recorded fetal heart rate to delivery; reduced proportion of births using oxytocin augmentation; and an increased proportion of women with severe hypertension receiving antihypertensive drugs. The numbers of stillbirths decreased 34% to 39 per 1000 total births (relative risk, RR: 0.66; 95% confidence interval, CI: 0.53–0.82), while newborns with an Apgar score of 1–5 halved to 28 per 1000 live births (RR: 0.53; 95% CI: 0.41–0.69).

Training 80 people over 2 days of the seminars costed approximately 800 United States dollars (US$), including laminated pocket guides (US$ 4.80 each), refreshments, photocopies and stationery. The same four whiteboard partographs were used at each seminar (US$ 45 each). A mannequin was already available when a neonatal resuscitation station was added in 2017. The intervention development and evaluation were led by a doctoral fellow and not included in these costs. We have not estimated opportunity costs for voluntary time spent by facilitators, seminar participants, study team members and external reviewers.

## Lessons learnt

Introducing locally-tailored clinical guidelines, with quarterly repeated training on their use, improved intrapartum care and birth outcomes in Zanzibar’s tertiary hospital, which appears to have been sustained for 4 years (Herklots T et al., Mnazi Mmoja Hospital, unpublished data, 2018). These changes occurred despite no improvements in staff numbers and staff turnover, no additional medical technology, no other training interventions and continual shortages in supplies. We found high motivation among many birth attendants to learn and to improve care, without additional financial compensation (Box 2).

Box 2 **Summary of main lessons learnt**

- Locally adapted clinical guidelines for intrapartum care, co-created with birth attendants, were associated with improvements in knowledge, skills, quality of care and perinatal outcomes.
- Evidence-based clinical guidelines produced at the international, regional and national levels may not lead to change if they cannot be easily adapted at facility level, taking into account resource constraints of particular settings.
- Short, repeated in-house training was highly acceptable among birth attendants, even without financial compensation for attendance outside working hours.

Nevertheless, developing the PartoMa guidelines was demanding on resources, requiring staff time and capacities, project funding and robust coordination of partners.^2^ As reported from Uganda,^3^ such resources can rarely be spared routinely in fragile health-care systems. While WHO encourages guideline adaptation, large-scale international, regional and national clinical guidelines should be better attuned to the constrains of low-resource settings, thereby facilitating facility-level adaptations. 

Maternal health guidelines targeting low-resource settings have typically relied on top-down development by experts, often without end-users’ feedback or pilot testing, followed by inflexible cascade implementation and no post-implementation evaluation.^1^^,^^3^^,^^12^^,^^13^ An example is WHO’s guide on managing complications in pregnancy and childbirth,^5^ which has informed multiple intervention programmes in low-resource settings, including eHealth solutions. Yet, according to a systematic literature review,^2^ few evaluations of the guide’s use and effects have been documented since its initial publication in 2000. 

When comparing multiple international clinical guidelines in maternal health, inconsistencies have been found across apparently high-quality, evidence-based guidelines.^1^ This emphasizes the limitations of experimental studies, and the strong influence of values, culture and professional traditions.^14^ Translating evidence into clinical recommendations inevitably requires judgements on benefits and risks, as well as the inclusion of evidence-weak recommendations when weaving together the evidence.^12^ We find the limited space given for end-users’ input in these processes concerning.^1^^–^^3^^,^^12^ Our experience was that such input from a low-resource maternity unit would include aligning guidance with the ratio of attendants to women in labour and with the local level of professional competencies; assessing the risks of contradictory recommendations or an overload of counterproductive guidelines; integrating basic and emergency management; and awareness of additional challenges faced by both providers intended to use the guidelines and the women in labour.

Supported by the study team, a steering group of volunteer birth attendants in Zanzibar have distributed guides and organized seminars since 2016. We have observed signs of ownership of the programme within the steering group, although there are particularly challenges to sustainability due to high staff turnover. In June 2018, the Zanzibar health ministry, with support from the United Nations Population Fund, took over the continuation and scaling-up of the intervention. The PartoMa seminars are now conducted quarterly in both of Zanzibar’s main islands. We are currently setting up a study in five maternity units in Dar es Salaam, United Republic of Tanzania, 'to assess whether the intervention can be easily context-adapted and effectively implemented at similar low-resource maternity units, and whether the process can serve as a model for other areas of health care. The PartoMa study has grown into a valuable single-setting analysis of the applicability of international guidelines targeting maternity care in low-resource settings. We acknowledge the ethical dilemmas of producing achievable guidance for use in settings with inadequate human and economic resources. However, we argue for decision-making support that assists birth attendants in saving lives, rather than best-evidence guidelines that are not used. The global community has called for context-appropriate implementation strategies with clinical guidelines formulated to facilitate understanding and use.^1^^,^^3^^,^^12^^,^^15^ Within and beyond maternal health, realistic clinical guidelines and associated training are conditional for strengthening health systems’ accountability and a fundamental right for health care providers being responsible for the lives of others. 
